# Lessons learned from England’s Health Checks Programme: using qualitative research to identify and share best practice

**DOI:** 10.1186/s12875-015-0365-z

**Published:** 2015-10-20

**Authors:** Hanif Ismail, Shona Kelly

**Affiliations:** Centre for Health & Social Care Research, Sheffield Hallam University, Sheffield, UK; Department of Health & Life Sciences, York St John University, York, UK

**Keywords:** Health checks, General Practice, Cardiovascular diseases, Early diagnosis

## Abstract

**Background:**

This study aimed to explore the challenges and barriers faced by staff involved in the delivery of the National Health Service (NHS) Health Check, a systematic cardiovascular disease (CVD) risk assessment and management program in primary care.

**Methods:**

Data have been derived from three qualitative evaluations that were conducted in 25 General Practices and involved in depth interviews with 58 staff involved all levels of the delivery of the Health Checks. Analysis of the data was undertaken using the framework approach and findings are reported within the context of research and practice considerations.

**Results:**

Findings indicated that there is no ‘one size fits all’ blueprint for maximising uptake although success factors were identified: evolution of the programme over time in response to local needs to suit the particular characteristics of the patient population; individual staff characteristics such as being proactive, enthusiastic and having specific responsibility; a supportive team. Training was clearly identified as an area that needed addressing and practitioners would benefit from CVD specific baseline training and refresher courses to keep them up to date with recent developments in the area. However there were other external factors that impinged on an individual’s ability to provide an effective service, some of these were outside the control of individuals and included cutbacks in referral services, insufficient space to run clinics or general awareness of the Health Checks amongst patients.

**Conclusions:**

The everyday experiences of practitioners who participated in this study suggest that overall, Health Check is perceived as a worthwhile exercise. But, organisational and structural barriers need to be addressed. We also recommend that clear referral pathways be in place so staff can refer patients to appropriate services (healthy eating sessions, smoking cessation, and exercise referrals). Local authorities need to support initiatives that enable data sharing and linkage so that GP Practices are informed when patients take up services such as smoking cessation or alcohol harm reduction programmes run by social services.

**Electronic supplementary material:**

The online version of this article (doi:10.1186/s12875-015-0365-z) contains supplementary material, which is available to authorized users.

## Background

The number of people in England and Wales suffering with cardiovascular disease (CVD) (which includes coronary heart disease, stroke, peripheral artery disease and aortic disease [1]) amounts to more than 3 million [2]. CVD is not only the most common cause of death accounting for 124,000 deaths (1 in 3 deaths in 2005) [2] but is also a major cause of illness and disability.

In 2009 the National Health Service (NHS) Health Check programme was launched in England at an annual cost of £250 million [3]. It is a cardiovascular risk assessment programme for all adults aged 40–74 years aimed at the general public, rather than people who already know they have CVD. It is intended to reduce known socio-economic and ethnic inequalities in cardiovascular health [4, 5].

The Health Check [3] involves systematic screening to measure CVD risk factors (e.g. smoking, obesity and blood pressure) and involves a set of lifestyle and behaviour questions and measurements (see Table 1 below for a complete list).

**Table 1:** Health Check components [3]

Age
Gender
Smoking status
Family history of coronary heart disease
Ethnicity
Body mass index (BMI)
Cholesterol level
Blood pressure
Physical activity level - inactive, moderately inactive, moderately active or active
Cardiovascular risk score
Alcohol Use Disorders Identification Test (AUDIT) score

Responses/measures are inputted into a risk calculator which generates a risk score. Scores that are in excess of 20 % are considered indicative of a high risk of developing CVD and result in further referral/recommendations for statins or as a minimum an annual appointment to discuss lifestyle advice and behaviour changes. Those below the 20 % threshold are offered lifestyle advice and recalled every 5 years. Department of Health (DoH) [6] modelling estimates that the programme could potentially prevent 650 deaths and 9500 non-fatal myocardial infarctions and strokes each year if 75 % uptake from the target population is achieved.

The Health Check was designed to be delivered in a variety of settings including general practices, pharmacies, work places and other community venues [7, 8] to make it accessible to as wide a population as possible. But, in 2009 when the programme was first introduced by the Primary Care Trusts (PCTs) that were tasked with delivering Health Checks to their local populations most PCTs elected to deliver Health Checks through primary care (known as General Practitioner (GP Surgeries) following evidence [9] that found screening by GPs as the most effective way of identifying unknown cases of CVD. Modelling from the DoH indicated that by the end of 2013, all GP practices in England should have invited every adult between the ages of 40 and 74, not already on a CVD register, to have their risk of developing a vascular disease assessed and managed.

However, the DoH does not seem to have compared predicted with actual uptake as audit data [10] shows that around 40 % of those that are eligible have not attended and these may be those most at risk. The danger is that if the Health Check fails to reduce vascular risk in those groups, who are most at risk of developing a vascular illness, it may not achieve its aims and may have the opposite effect of increasing health inequalities [11].

The responsibility for delivering Health Checks changed in April 2013 as there was devolution of NHS management to regions and PCTs were abolished. Local authorities [12] were assigned responsibility for the organisation and delivery of the national Health Check Programme as part of their public health remit to prevent ill health [13].

While a number of quantitative evaluations [14–17] of the Health Checks have been undertaken these have primarily focused on attendance, uptake and outcomes in terms of statin prescribing and changes in lifestyle. Importantly, considerable variations in recruitment rates have been found. Few qualitative studies have been undertaken exploring the views of those involved in providing Health Checks or highlighting good practice doing so.

This study reports on findings from Local Authority commissioned qualitative studies that were conducted in GP practices with the intention of identifying best practice and explore barriers within GP Surgeries that are inhibiting uptake of the Health Checks invitation

## Methods

The study was carried out in three sites across the Yorkshire region of the UK (see Tables 2 and 3 for details). We began with an evaluation in 2010 of a pilot of the Health Checks Programme site (funded by the National Institute of Health Research) followed by research in two further sites that had established programmes after the full rollout of the Programme (contracted evaluations for the Local Authorities).

**Table 2:** Details of participating practices

	Site A	Site B	Site C
Number of practices	5	10	10
Total number (range of interviews)/site	13 (2–5)	23 (1–3)	22 (2–3)
Range of patient population size of the practices	2819–13,903	1117–10,459	1387–18,960
Deprivation (pentile number)			
1st (most)	0	4	3
2nd	4	4	3
3rd	0	2	3
4th	0	0	1
5th (least)	1	0	0
% invitations taken up (range)	pilot	60–70 %	29–93 %

**Table 3:** Staff involved in delivering Health Checks

	Site A	Site B	Site C
Health Care Assistants (HCAs)	6	13	11
General Practitioners (GP)	2	2	1
Practice Managers (PM)	1	3	5
Practice Nurses (PN)	4	5	5
Total no of interviews	13	23	22

We conducted in depth qualitative interviews and obtained written informed consent for participation in the study prior to commencement. Discussions were facilitated by a topic guide and included personal and organisational level preparation, impact on workload, barriers to delivering the Health Check, support requirements and factors that have led to successful uptake or have presented challenges and led to difficulties in patient recruitment. Patients were also interviewed in the pilot site and this has been reported elsewhere [18].

The two local authority reviews had slightly different remits. Site B elected to focus on identifying the factors associated with successful recruitment into the Health Check Programme where success was classified as more than 60 % uptake. Site C focused on both highly successful and very unsuccessful programmes to ensure that the factors identified in successful sites were indeed not present in unsuccessful sites and that differences identified weren’t due to some other factor.

### Sampling and recruitment

This paper reports on the perspectives of staff across all three sites using a qualitative design involving analysis of in-depth interviews (see Table 3) including: Health Care Assistants (HCAs), General Practitioners (GP), Practice Managers (PM), Practice Nurses (PN) and other support staff. We collected in-depth interview data from 58 staff involved in the delivery of the Health Checks working in 25 GP practices. An advisory team composed of academics and Public Health representatives from each Local Authority was assembled to guide the research.

### Ethics

Research ethical approval was granted for the pilot study in August 2011 by East Yorkshire & North Lincolnshire Research ethics Committee (ref: 10/H1304/22). Formal ethical approval was not required for the other two studies as they were classed as commissioned service evaluations, however, Sheffield Hallam University approvals were sought and granted in 2013/14.

### Topic guide development

A semi structured interview guide (see Additional file 1) was developed based upon the aims of the study and review of the relevant literature, this was tested and further adjustments were made after a piloting phase. The topic guide began with questions around the understanding of the Health Check, role of staff in the delivery of the checks, training received, and process of delivering checks, commitment to the programme and identifying factors that led to increased uptake.

### Interviews

Staff, were interviewed about their perceptions of the programme and the challenges they face in the delivery of the Health Checks. We contacted practices to seek their permission to participate and determine which members of staff were involved in the delivery of the Health Check. Interviews took place at work and all participants gave their verbal permission for the interviews to be audio taped. Duration of interviews ranged from 25 to 45 min.

### Data analysis

Full transcripts from the interviews were produced for analysis. The data were analysed systematically by the team using the framework approach [19] which involved detailed familiarisation with the data, identification of key themes and interpretation of the findings within the context of other research and practice considerations.

We began by working within cases to devise an index of key concepts and themes drawing on ‘a priori’ issues linked to the research objectives. We followed this process by making comparisons across cases, refining themes and developing a framework. We then applied themes systematically to the data through coding and rearranging according to the thematic framework. Indexed data was then transferred to a grid to compare cases and identify similarities and differences, finally we produced illustrative charts and used mapping and interpretation to record the nature and scope of the themes to develop associations. The emerging analysis was thematic and iterative and to ensure reliability [20] was analysed independently by two colleagues that were not involved in the data collection process and discussed with the advisory group.

The findings from the analysis presented below identified six main inter-related themes: invitation to attend, awareness-raising, barriers to behaviour change, organisational barriers, training requirements, effective team working, and benefits and drawbacks of Health Checks.

## Results

In total we contacted 41 GP practices and managed to recruit 25 practices (see Table 1 for details).

In terms of recruitment, for site A we wrote to all seven GP practices that were chosen by the local (PCT) to pilot the Health Checks and five agreed to participate. For sites B & C we sent a flyer to all GP practices in the area informing them that an evaluation of the Health Check was being conducted and that they may be approached and asked to participate. With guidance from the project advisory team we targeted 35 GP practices asking them to participate. These GP practices were chosen to reflect diversity in terms of performance benchmarking and key performance indicator targets set by the Department of Health [21]. We contacted 12 practices from site A (three top performing, three mid performing and three under- achieving) and managed to recruit ten. In site C we also contacted 12 practices but were only able to recruit five, we contacted a further ten practices and from these we managed to recruit a further five.

### Invitation to attend

For a health screening programme to be effective, clear information needs to be provided about what the screening entails, possible benefits and potential harm identified so that patients are empowered to make an informed choice to attend [22, 23]. GP practices adopted a number of different approaches to inviting patients for Health Checks, through letters, telephone calls and opportunistically while patients were in the practice for an unrelated appointment. Several interviewees stressed the importance of the existing relationships they had with patients and the fact that they knew the type of approach that would work best with their population.

“*If we’re struggling with hard core patients - we know who they are as we are a small practice and know these families for generations, and there always are some in every practice, we have somebody telephoning them*” [PN- Site C practice 3]

“*The good ones, the good kids I call them will come in and make an appointment; the naughty children won’t. So what we need to do is think right the people that have had the letters but they haven’t made the appointment, that then is my second hit list where I’m going to get a receptionist, possibly one of my administration team, to come in for a day and sit there with a phone*” [PM-Site A practice 2]

In terms of recruitment the most successful practices described a generally flexible proactive approach to encouraging people to come for the tests that included early mornings or extended opening hours:

“*I mean we have the healthcare assistants are here at 8:30, so they can have early bloods done before they go to work, and the nurse can see them before they go to work, so we’re trying to offer those facilities to people to catch them.*” [HCA- Site B practice 8]

“*I tend to get a lot on a Monday morning, the men that are working, on their [way] to work, because I start at seven you see and it’s always booked, always.*” [PN-Site A practice 3]

However, two restricting factors were mentioned that affected their ability to be completely flexible. The first was the need for a fasting blood test, although at least eight of the practices had forfeited this for, only slightly less accurate, onsite patient testing and the second was the need to have at least two appointments – one for the blood tests and one for the results:

“*We went for an HbA1c* [blood test which measures blood glucose levels retrospectively over the past 8–12 weeks] *rather than a glucose because we thought people can’t be faffed with fasting bloods, that’s the sort of thing that puts people off. It’s the two appointments I think that puts people off*” [GP- Site B practice 1]

“*It’s the two appointments I think that puts people off. I do because if you’re working you’re coming twice, but we need the bloods to be able to say to people right we’ve analysed your bloods and blah, blah, blah, so I don’t see how we’re going to do anything about that.*” [HCA- Site C practice 7]

### Awareness-raising

Most interviewees mentioned that there was a lack of awareness of the Health checks and that there needed to be messages in the media to raise awareness.

“*Perhaps it might be an idea if they run a national campaign about CVD go to your doctor if you’re in this age group.*” [HCA- Site A practice 4]

“*You know like flu vaccines, it’s advertised ain’t it? It’s advertised nationally, chemist, supermarkets, TV, wherever, and I don’t think NHS Health Checks, they call them CVD risk assessment, what the hell is a CVD, patients don’t know, we did an audit, people didn’t know what it was. So you’re ringing them up, oh would you like to come for a CVD cardiovascular, you know, what is it, what does that involve?*” [PN- Site C practice 7]

We found a wide range of opinions when we asked practice staff about the types of patients that do not attend appointments. Some GP practices felt that older people were less likely to attend as they were resistant to change. Others thought younger people didn’t turn up because they were less concerned about their health; some mentioned working people while others cited the unemployed. However all agreed that people from hard to reach groups like the homeless, travellers, drug users and black and minority ethnic groups (BME) were less likely to attend. Language and cultural issues were mentioned by a number of staff as a major barrier in engaging with BME groups:

“*I think the withdrawal of the translators has been a big issue. Because we had a Slovakian translator and one with the ethnic minorities as well, for the Black and Asian community, and they were both pulled eighteen months, two years ago, due to funding.*” [PN- Site B practice 3]

Crucially the reason for non- attendance was not always solely due to language barriers – one of the interviewees said that they felt the problem was more deep-seated:

“*It’s the Latvian and Baltic States that I’d say and particularly the Roma population, really there are big issues about their sense of health awareness, and the idea of doing something to prevent something is a difficult idea to get over.* [GP- Site C practice 4]

“*I think Asian women are the hardest group to engage with probably due to cultural differences and the fact they have so many family responsibilities on their shoulders..looking after 3 generations in 1 house plus the cooking cleaning and caring…means their own health and well-being always come last!*” [GP- Site B practice 7]

### Barriers to behaviour change

Coming up with ways of inviting patients in and encouraging them to come to the appointments was mentioned as being only a part of the equation, the most important aspect was facilitating effective behaviour change. Interviewees reported that due to lack of resources they were unable to engage with patients on an individual basis and provide support to keep them motivated enough to adopt long-term diet and lifestyle changes that would make a difference to their lives.

“*We used to have things called exercise referral and we refer people to free gym sessions and send them to Slimming World and they’d get Slimming World sessions. We had really good responses and really good uptake for that, but that’s all gone now. It’s just Change 4 Life* [Government led social marketing campaign to reduce obesity]*; a lot of people don’t want to go on that. They were much more willing to go and to say join Slimming World which they’ve heard of before and because they pay for it normally and they’re going to get it for free, or go to the gym which they normally pay for but would get it for free; they’re much more likely to take that on.*” [PN- Site B practice 6]

“*They don’t do [exercise referrals] anymore. They have stopped doing that. It is just people talking about diet which is a bit boring. Because we had a twelve month contract with the gym and they loved it*”. [PN- Site C practice 1]

This practice nurse, in an effort to replace the exercise referrals, had on her own initiative and in her own time, started a walking group for patients.

A second barrier suggested was that perhaps some lifestyle changes need a household-approach rather than an individual approach:

“*Even if you access them, even if you find out that they’re a really high risk score then getting these people to take on board you know the lifestyle changes, changes to their diet, exercising more. It’s very difficult to get them to take those changes on; it’s so kind of ingrained in their culture. Their family life and everybody in their family smokes, they all eat unhealthily. It’s very difficult to educate these people. I don’t think we’re set up in primary care to do that you need people more in the community based going into people’s homes and talking to a whole family. There’s no point in talking to a husband if the wife cooks the food. Sometimes you’ll say bring the wife in if she cooks all the food but if you don’t address the wife as well then it’s not going to make any difference is it*” [PN- Site A practice 3]

### Organisational barriers

While funding was mentioned as a general limiting factor by all the study participants, there were also some quite specific barriers. Staff were facing a number of practical/ structural barriers (see Table 4) that impacted on their ability to provide an effective service.

**Table 4:** Practical barriers in delivering the Health Checks

Staff time/hours	“*We haven’t had the, I don’t think we’ve had the HCA funding to be able to do that quite honestly; to start offering sort of afterhours and things like that* [PN]
Space in the building	“*There’s not enough rooms! You know, it’s not a case of getting clinicians in, we can get them in, but we’ve nowhere else to put them.*” [PN]
Software	“*I print off the list of patients from and I trawl through them, looking, making sure that they’re all within that bracket, because there’s some that are underage (under 40) and some that are over the age (over 74), so it’s a pain, filtering those patients out, 19-year-olds and 90-odd-year-olds on there*”. [HCA]
Leaflets and posters	“*I don’t think we’ve got any* [leaflets] for the *Health Checks. And I think they’re hard to get hold of actually, but I don’t think we send those out either. But again if they rejigged some posters and leaflets and things to get people’s interest again, you know like they do every year for flu jabs?*” [PN]

### Training requirements

The health practitioners interviewed all had different levels of work experience ranging from 12 months to 25 years. There were a range of comments about the support given to them to undertake Health Checks. Support was either provided through PCT led training and briefings, provided by GPs in their practice, or from a senior nurse. Training was suggested, by most of the participants, as being an area in which work needed to be undertaken. Around a quarter of respondents (15) said that they had absolute gaps in their knowledge. Interestingly, these were all health care assistants who, relative to other staff across the NHS, receive very little training, although they make up 40 % of the workforce and are allocated only 5 % of the national funding budget for training [24]

“*It [training on risk factors] would be helpful actually because then you’ve got more knowledge on, you know when somebody does ask you that, because there’s the odd patient that will come back and then I have to go to the nurse or have a word with the GP and say, look, they’ve asked me this question, what do I say to them? Because I don’t want to step over the line*” [HCA- Site B practice 10]

“*[Training] would be good. As I say, we just learnt from our healthcare assistant what to do; basically it was like kind of on the job training… It would be nice to understand it in depth more, wouldn’t it?*” [HCA- Site C practice 7]

### Effective team working

A reoccurring theme from a number of the interviewees placed the responsibility for the success of the Health Check campaign firmly with their staff team:

“*We have really, really good staff in this practice. X is an excellent nurse, X healthcare assistant, really good. She’s a* really proactive person” [PM- Site B practice 2]

“*We’ve got four practice nurses and two healthcare assistants and we work really closely together as a team doing this. We do some of the initial health checks if the health care assistants are busy but they tend to do most of it. We tend to see the high risk patients when they’re coming back but [name removed] has devised a list, a sheet that we put everybody down on who we see and this is a way that we can score. So the blood results come back to the nurses, we review the blood results and then do the risk scoring thereafter its a slick operation and its only possible cause we have such a good working relationship*”*.* (PM- Site A practice 5)

Although several factors were cited by the interviewees as the likely reason that the team were effective, two key factors stood out. The first was the defined roles of the team members and the manner in which they worked well as part of a cohesive team. Each successful practice could identify which person had a particular role – for example, who would run the computer searches, make the initial invite letter/phone call, send reminder contacts, undertake the blood tests, update the system, run the risk scoring, contact the patients, carry out the follow-ups. These were named individuals (as opposed to, for example, the HCA who happened to have spare time) that were part of a defined team with a named individual having overall responsibility for the campaign.

The second attribute of the successful team encompassed the particular character of the individual or team which means that they went above and beyond the immediate roles/needs of the campaign. While this is not a tangible attribute the example below, highlights the effectiveness of this approach:

“*I always ask nurses what they do because I think it’s better to know what they do, because you can’t offer somebody it if you don’t know the background to it. I think anyway so, then when they ask questions you’re not like, I don’t know. So I just like to find out what it’s all about and then you can pass that information on really*”. (PM- Site C practice 8)

### Perceptions of the benefits and drawbacks of Health Checks

Although there was a general consensus, that the Health Checks had the potential to be extremely beneficial in terms of early detection and prevention of chronic diseases. Some views were more critical and doubtful of the impact of the programme (see Table 5).

**Table 5:** Health practitioners’ views of the Health Checks programme

Positive	Negative
People are checked earlier in life rather than waiting for problems to develop	The worried well are most likely take the messages on board
Help to identify people at high risk that had been missed before	The Practice is good at screening for high risk patients even without the formal Health Check
Help to identify and support people with high cholesterol, high blood pressure or diabetes, as well as heavy smokers and heavy drinkers	It has a big impact on workload and cost implications
Many people wanted a cholesterol check.	There is uncertainty about whether people take the advice given to them in Health Checks
Those that attended the health check were motivated and open to advice.	They get the patients who want to attend but not the patients who need their intervention the most
The Health Checks have worked well for the worried well as well as those that have a high risk	They are not getting full time workers as appointments are only offered before 4 pm
Good patient feedback	Appropriate referrals cannot be made due to cuts in services

One interviewee (General Practitioner) in particular was not entirely convinced about the evidence cited for the effectiveness of the Health Check which seemed to contradict much of what he had been taught about population-level screening being cost ineffective and was clearly an advocate for a more targeted approach:

“*I think really this is mass screening and there’s not a great deal of proof behind it, but it did seem like it was stuff that we were kind of largely doing anyway. We were always pretty good at offering risk assessment at new patient checks. Not entirely convinced with being told we have to offer a check to everyone. It’s something I’m quite passionate about targeting those most at risk but I think certainly there are others within the practice that would be as well. We’ve always seen ourselves as quite forward thinking from that point of view*” [GP- Site A practice 3]

## Discussion

This unique qualitative study has highlighted issues in terms of delivery of the Health Checks programme from the practitioner’s perspective. The results indicate that while the Health Check is perceived as a worthwhile exercise to identify patients at risk of CVD a number of issues around organisation and structural barriers were identified. Importantly we found that there appeared to be no ‘one size fits all’ blueprint for success, rather a variety of different measures and approaches that lead to successful uptake.

The key components present in all GP practices that had done well included adoption of an approach that had evolved and developed over time in response to local needs to suit the particular characteristics of the patient population. In addition, individual staff characteristics came into play. For example being proactive, enthusiastic and having specific responsibility as well as having a supportive team all facilitated participation. Training was clearly identified as an area that needed addressing and practitioners could clearly benefit from CVD specific baseline training and refresher courses to keep them up to date with recent developments in the area. However there were other external factors that impinged on an individual’s ability to provide an effective service, some of these were outside the control of individuals and included cutbacks in referral services, insufficient space to run clinics or general awareness of the Health Checks amongst patients.

While the Health Checks programme has been running for more than 4 years and should in theory be well embedded in General Practice, a number of the issues identified by this study are still very much evident. A particular point of concern is that we saw little differences in the views of practitioners from our pilot site study which was undertaken in 2009 in comparison with the more contemporary studies that were undertaken in 2014. We found that there was a distinct sense that practices were largely working in isolation from each other and not sharing good practice in terms of innovation and process development. This finding is not surprising given that after start-up the Health Checks Programme offers no further baseline training for new staff or refresher courses which this study demonstrates are needed.

Although the recent changes in the delivery of primary care services have seen responsibility shift to local authorities it remains to be seen if they will critically evaluate delivery at the practice level with a focus on longer-term outcomes and introduce the necessary changes to increase uptake.

A positive outcome of the evaluations has been that Site B has taken on the key learning outcomes and incorporated them into practice. The commissioning body (site B) has enthusiastically taken up the findings from their evaluation and created an information package for the participating GP Practices and has also held an information day which was attended by the head of the Health Checks Programme. During this training day the participants signed up for skills workshops on alcohol harm reduction, information and brief advice and smoking cessation. Evidence from this research was used to create information posters, patient information leaflets, advertising coasters and scratch cards that patients could use to confirm that they are eligible for the programme. The Local Authority is monitoring Health Checks uptake to evaluate the effect of this initiative but in feedback on the day all the attendees commented that this refresher was exactly what was needed.

### Study strengths and limitations

In terms of study limitations although this was a modest scale qualitative study carried out in three cities we reached saturation and believe that we interviewed a sufficient number of staff, and that our sample was representative of the wider population. Recognising we only recruited practices from site B that were the most successful in terms of patient recruitment we compensated for this by ensuring we recruited sufficient numbers of low performing practices from site C to confirm the findings.

A particular strength of the paper is that the sites encompassed a range of different GP practices in terms of size, deprivation and ethnic composition and levels of experience in conducting Health Checks. Additionally we sought the views of a wide range of practitioners from health care assistants to GP’s.

### What this study contributes?

Although a number of quantitative evaluative studies [14–17] have been undertaken looking at uptake there is a paucity of research looking at the qualitative experiences of those delivering Health Checks. This study readdressed the balance by exploring issues from the perspective of practitioners. The results build on the body of survey evidence provided by Nicholas et al. [25] and concurs with the McNaughton et al. [7] study which highlighted a number of similar issues arising in the delivery of Health Checks particularly around staff training.

## Conclusion

This study highlights the everyday experiences of practitioners in terms of the facilitators and barriers they face in delivering Health Checks. We know that GP practices have had a high degree of autonomy in organising and running the Health Check programme and this is an advantage for identifying successes and barriers to recruitment. Practices that had strong leadership, clearly defined roles and the freedom to customise their approach to suit their work and staff structure, clinical space, and knew the characteristics of the community in which they were based were more successful in terms of patient recruitment. Our findings indicate that training at all levels (baseline or refresher) is required for staff so that they are able to understand the individual components of the Health Check and can explain the importance of CVD screening to patients. There also needs to be clear referral pathways so staff can refer patients to appropriate services such as healthy eating sessions, smoking cessation, and exercise referrals. Local Authorities also need to support initiatives that enable data sharing and linkage so that GP Practices are informed when patients take up services such as smoking cessation or alcohol harm reduction programmes run by social services.

## Additional file

Additional file 1:
**Health Check Evaluation project.** (DOCX 16 kb)

## Abbreviations

NHS: National Health Service
CVD: Cardiovascular disease
DoH: Department of Health
PCTs: Primary Care Trusts
GP: General Practitioner
HCAs: Health Care Assistants
PM: Practice Managers
PN: Practice Nurses
