# Supplementary material for: Lessons learned from England’s Health Checks Programme: using qualitative research to identify and share best practice
Source: BMC Fam Pract. 2015 Oct 20;16:144. doi: 10.1186/s12875-015-0365-z (PMC4618054; doi:10.1186/s12875-015-0365-z)
Supplement: Additional file 1: — Health Check Evaluation project. (DOCX 16 kb) [file 12875_2015_365_MOESM1_ESM.docx]

**Additional file 1**

**Health Check Evaluation project**

**INTERVIEW SCHEDULE**

1. **Personal role, and your views and experiences**
   - 1. **Can you tell me about your role and how it fits in with the health check programme in your practice?**
        1. How and why did you become involved in the programme?
        2. What are your opinions on how the health check programme is delivered in this practice?
           1. What works well?
           2. What might be improved?
        3. Have there been any changes in how you have been involved in the programme over time?
           1. If yes, why and in what way? Do you think these changes have improved/detracted from the delivery of the checks at your practice?
     2. **When you first became involved in the health check programme, did you have some understanding of the programme or have you had to learn and develop along the way?**
        1. Have you received any training relevant to your role in the programme?
           1. If yes, from whom and was the training useful / timely / unhelpful – can you explain?
           2. Can you think of any further training that would improve your delivery of the health checks?
           3. How competent do you feel in your knowledge of behaviour change methodologies such as Motivational Interviewing
           4. How confident to you feel in practicing behaviour change methodologies such as motivational Interviewing.
     3. **In addition to any training, have you received any additional support for your role in the programme from within or outside of your practice?**
        1. From who and how often?
        2. Do you have to search out support or is it provided / offered routinely?
        3. Has support been generally useful / lacking and if so, can you explain?
        4. Is there any additional support you may require? - How do you think this would improve the delivery?
     4. **Talk me through the health check process in this practice**
        1. How do you ensure that health checks are delivered in accordance with the national guidance?
        2. How are people invited? Do you send letters/telephone calls/opportunistic (during unrelated surgery visits / appointments)
        3. Does your practice do any general awareness raising around health checks
        4. How are results communicated to patients? Do patients act on them? In what ways?
        5. Do results lead to behaviour change interventions? How
     5. **Talk me through the process for ‘high score’ patients**
        1. For example advice, follow up, reminders, discuss at meetings, refer to other services / clinics within / outside of practice
     6. **Do you routinely monitor take up of invitations?**
        1. If not you, is this someone else in the practice’s responsibility?
2. **Commitment to the programme**
   - 1. **What are your views and expectations of the programme for individual patients?**
        1. Do you think the checks will identify individual patient’s problems earlier/ more effectively or no differently than what was routine / standard practice before the programme?
     2. **How does the practice target at risk populations/individuals/hard to reach patients?**
        1. Do you think the programme has a positive or negative effect on inequalities in health in this practice area?
        2. What is the approach used to discuss health checks in your practice (both high scores and health checks generally)?
           1. e.g., in some practices, health checks are a routine agenda item at practice meetings, whilst in others they are discussed as and when necessary.
     3. **Learning and changes**
        1. Do you have any examples about how your approach (or your practice’s approach) to health checks has changed as a result of patient feedback, personal experience or practice discussions?
           1. Perhaps about how patients are invited or followed up? Or how non-attenders/target populations are approached or encouraged?
3. **Factors for success**
   - 1. **Can you identify some key lessons from your involvement in the health check programme?**
        1. What do you think has worked well/Not so well?
        2. What could be improved?
     2. **Is there anything else you would like to add?**
